# Incorporation of CENP-A/CID into centromeres during early *Drosophila* embryogenesis does not require RNA polymerase II–mediated transcription

**DOI:** 10.1007/s00412-022-00767-2

**Published:** 2022-01-11

**Authors:** Samadri Ghosh, Christian F. Lehner

**Affiliations:** Department of Molecular Life Sciences, University of Zurich, Zurich, Switzerland

**Keywords:** Centromere transcription, Centromere chromatin, CENP-A/CID deposition, Alpha-amanitin, Triptolide

## Abstract

In many species, centromere identity is specified epigenetically by special nucleosomes containing a centromere-specific histone H3 variant, designated as CENP-A in humans and CID in *Drosophila melanogaster*. After partitioning of centromere-specific nucleosomes onto newly replicated sister centromeres, loading of additional CENP-A/CID into centromeric chromatin is required for centromere maintenance in proliferating cells. Analyses with cultured cells have indicated that transcription of centromeric DNA by RNA polymerase II is required for deposition of new CID into centromere chromatin. However, a dependence of centromeric CID loading on transcription is difficult to reconcile with the notion that the initial embryonic stages appear to proceed in the absence of transcription in *Drosophila*, as also in many other animal species. To address the role of RNA polymerase II–mediated transcription for CID loading in early *Drosophila* embryos, we have quantified the effects of alpha-amanitin and triptolide on centromeric CID-EGFP levels. Our analyses demonstrate that microinjection of these two potent inhibitors of RNA polymerase II–mediated transcription has at most a marginal effect on centromeric CID deposition during progression through the early embryonic cleavage cycles. Thus, we conclude that at least during early *Drosophila* embryogenesis, incorporation of CID into centromeres does not depend on RNA polymerase II–mediated transcription.

**Supplementary Information:**

The online version contains supplementary material available at 10.1007/s00412-022-00767-2.

## Introduction

Centromere function is essential for error-free chromosome segregation during mitotic and meiotic divisions. In animals, centromere identity is usually specified epigenetically (Mellone and Fachinetti 2021). The epigenetic marking of centromeres is mediated by special nucleosomes, which contain a centromere-specific histone H3 variant instead of canonical H3. The human centromere-specific histone H3 variant was designated as centromere protein A (CENP-A) (Earnshaw and Rothfield 1985; Palmer et al. 1987) and the orthologous protein of *Drosophila melanogaster* as centromere identifier (CID) (Henikoff et al. 2000). CID is required and sufficient for specification of centromere identity (Blower and Karpen 2001; Mendiburo et al. 2011; Olszak et al. 2011; Palladino et al. 2020; Roure et al. 2019), and similar evidence has been obtained in other species (Mellone and Fachinetti 2021; Murillo-Pineda and Jansen 2020). Therefore, the deposition of CENP-A/CID into chromatin during progression through the cell cycle must be controlled so that the epigenetic centromere mark is neither lost nor established ectopically. Considerable progress has been made in the analysis of the molecular mechanisms of CENP-A/CID loading and its control (Mellone and Fachinetti 2021). Studies with human and *D. melanogaster* cell lines have revealed extensive similarities. Centromeric chromatin does not contain a single contiguous stretch of CENP-A/CID nucleosome. Rather, blocks with CENP-A/CID nucleosomes are interspersed with blocks of chromatin containing canonical H3 nucleosomes (Blower et al. 2002; Sullivan and Karpen 2004). When centromeric DNA is replicated during S phase, it is accompanied by the distribution of the pre-existing CENP-A/CID nucleosomes onto the newly replicated sister centromeres (Jansen et al. 2007; Zasadzińska et al. 2018). Loading of additional CENP-A/CID does not occur in parallel with centromere DNA replication during S phase (Bobkov et al. 2018; Jansen et al. 2007; Lidsky et al. 2013; Mellone et al. 2011; Schuh et al. 2007; Shelby et al. 2000). However, nucleosomes containing histone H3 are deposited onto the newly replicated centromeres during S phase as “placeholder” nucleosomes (Dunleavy et al. 2011). Eventually, during early G1 of the next cell cycle, new CENP-A/CID nucleosomes are loaded into the centromeric regions, apparently replacing the placeholder nucleosomes (Bobkov et al. 2018; Jansen et al. 2007; Lidsky et al. 2013). Although human and fly cell lines share these characteristics of centromeric CENP-A/CID propagation during cell cycle progression, their loading machinery does not appear to be strictly homologous. *D. melanogaster* seems to use only a single assembly factor CAL1 (Chen et al. 2014; Erhardt et al. 2008; Medina-Pritchard et al. 2020; Roure et al. 2019; Schittenhelm et al. 2010), instead of the two factors HJURP and Mis18 complex that are conserved from yeast to humans (Müller and Almouzni 2017; Pan et al. 2019). Moreover, stage- and cell-type specific variation concerning the timing of CID loading during the cell cycle and also with regard to the symmetry of CID loading onto sister centromeres have been exposed by analyses in the *D. melanogaster* organism (Carty et al. 2021; Dattoli et al. 2020; Del García et al. 2018; Dunleavy et al. 2012; Ranjan et al. 2019; Raychaudhuri et al. 2012; Schuh et al. 2007). In early *D. melanogaster* embryos, where cell cycle progression is extremely rapid, CID loading was observed to start already during anaphase (Schuh et al. 2007).

Beyond specific assembly factors, CENP-A/CID loading appears to involve transcription of centromeric DNA (Corless et al. 2020; Liu et al. 2021; Mellone and Fachinetti 2021). The regional centromeres of mammalian and *Drosophila* chromosomes, as well as their flanking regions are comprised of highly repetitive, satellite-rich DNA sequences. Therefore, centromeric and centromere-proximal sequences are not yet part of reference genome assemblies. However, long-read single-molecule sequencing in combination with immunoprecipitation and fiber imaging of CID chromatin has largely clarified the DNA sequences present in the CID containing centromeric regions of the five *D. melanogaster* chromosomes (chrX, Y, 2, 3, and 4) (Chang et al. 2019). While pericentromeric DNA is composed primarily of satellites, centromeric CID chromatin includes complex DNA sequences with retroelements (Chang et al. 2019). The DNA sequence most strongly enriched in CID chromatin in all five centromeres is that of a particular retroelement, *G2/Jockey-3* (Chang et al. 2019). Interestingly, at least some of the centromeric *G2/Jockey-3* elements appear to be transcribed in *D. melanogaster* embryos (Chang et al. 2019). In cultured human and *D. melanogaster* cells, centromeric sequences are transcribed by RNA polymerase II (Bobkov et al. 2018; Bury et al. 2020; Chan et al. 2012; McNulty et al. 2017; Quénet and Dalal 2014; Rošić et al. 2014). Evidence for a functional significance of centromere transcription in the loading of centromere-specific nucleosomes has been obtained in a range of species (Corless et al. 2020; Liu et al. 2021; Mellone and Fachinetti 2021). An elegant demonstration of a functional interaction between transcription and CID deposition in cultured *D. melanogaster* cells involved a lacO/lacI system, permitting CAL1-GFP-LacI-mediated CID loading onto a chromosomal lacO repeat array (Chen et al. 2015a). This CID loading was shown to depend on recruitment of RNA polymerase II and transcription of the lacO array. Additional work reported the microscopic colocalization of the elongating form of RNA polymerase II and nascent RNA at endogenous centromeres (Bobkov et al. 2018). Moreover, stable assembly into centromeric chromatin after experimentally induced release of a CID variant protein from cytoplasmic retention was shown to be inhibited by concomitant short-term application of an inhibitor of RNA polymerase II (Bobkov et al. 2018). Overall, the work with cultured *D. melanogaster* cells provides strong support for a dependence of CID loading on centromeric transcription. Centromeric transcription was proposed to be required for eviction of the placeholder nucleosomes, allowing a subsequent incorporation of CID nucleosomes (Bobkov et al. 2018, 2020; Chen et al. 2015a).

A mechanism for propagation of centromere identity during cell cycle progression that depends on centromeric transcription cannot be used during early embryogenesis of *D. melanogaster*, if transcription is absent during these early stages. Absence of transcription during the earliest stages of embryogenesis is considered the norm in animals, as early development relies on maternally derived RNA and protein stores in the oocyte (Vastenhouw et al. 2019). Timing and dynamics of transcriptional activation of zygotic gene expression vary in different species. In mammals like humans and mice with relatively small eggs and slow initial cell cycles, the onset of zygotic transcription occurs already during interphase of the second cleavage cycle. In contrast, in large eggs with rapid early cell cycles, as in many invertebrates and non-mammalian vertebrates, high-level transcription begins at later stages. In *D. melanogaster*, embryogenesis starts in the characteristic, insect-specific manner. After duplication in S phase, chromosomes are separated during mitosis without cytokinesis, resulting in a syncytium. Progression through alternating S and M phases proceeds without intervening gap phases, at very high speed and in synchrony in all nuclei of the syncytial embryo. The mean duration of a nuclear cycle (NC) is only 8 min initially. Starting with NC9, a gradual slowdown occurs. The centripetally migrating nuclei reach the egg periphery during NC10, marking the onset of the syncytial blastoderm stage. After progression through NC11-13, the peripheral layer of nuclei is converted into a single-layer, cellular epithelium during interphase of NC14. Shortly after cellularization, which generates the cellular blastoderm, gastrulation starts 3 h after fertilization and egg deposition. Absence of transcription in early *D. melanogaster* embryos was suggested initially by experiments involving labeling of permeabilized or injected embryos with radioactive RNA precursors (Anderson and Lengyel 1979, 1980; Edgar and Schubiger 1986; Zalokar 1976). Moreover, genes that are clearly required zygotically for normal progression through the initial cleavage cycles do not exist (Merrill et al. 1988; Wieschaus and Sweeton 1988), except for a few cases with mild mutant phenotypes (Ali-Murthy et al. 2013; Ali-Murthy and Kornberg 2016). A recent, genome-wide analysis by RNA-Seq after labeling of newly synthesized RNA confirmed the gradual activation of zygotic gene transcription during the syncytial stages (Kwasnieski et al. 2019), in general agreement with earlier transcriptomic analyses (Ali-Murthy et al. 2013; Bosch et al. 2006; Lott et al. 2011; Renzis et al. 2007) and in situ hybridization studies with probes for selected genes (Ali-Murthy and Kornberg 2016; Erickson and Cline 1993; Pritchard and Schubiger 1996). Zygotic transcripts generated before the syncytial blastoderm stage are derived from only very few, short, and intronless genes, primarily involved in sex determination. While short interphases in combination with transcriptional abortion in M phase limits the accumulation of mature transcripts in early embryos (Kwasnieski et al. 2019; Rothe et al. 1992; Shermoen and O’Farrell 1991; Strong et al. 2020), regulation beyond NC duration inhibits zygotic transcription during the initial syncytial stages (Edgar and Schubiger 1986; Pritchard and Schubiger 1996). Absence or very low transcriptional activity during the initial cleavage cycles was also observed for non-polyadenylated RNA and transposable elements (Kwasnieski et al. 2019).

Beyond the analysis of zygotic genome transcription, results obtained with pharmacological inhibitors also appear to disfavor that CID loading during early embryogenesis depends on centromeric transcription. Injection of alpha-amanitin during NC5, at concentrations blocking transcription by RNA polymerase I and II, does not interfere with progression through the syncytial NCs (Edgar and Datar 1996; Edgar and Schubiger 1986; Gutzeit 1980). Similarly, triptolide, another potent inhibitor of transcription (Bensaude 2011; Chen et al. 2015b; Henriques et al. 2013; Titov et al. 2011; Vispé et al. 2009), is compatible with progression through the syncytial NCs (Hug et al. 2017). However, these observations cannot rigorously exclude an essential involvement of centromeric transcription during early embryogenesis for the following reason. At least in human cells, the normal level of centromeric CENP-A was found to be far above the amount required for centromere function during mitotic proliferation (Fachinetti et al. 2013; Liu et al. 2006). After acute recombinase-mediated CENP-A gene elimination, centromeric CENP-A protein was observed to decrease by 50% during each subsequent cell cycle. However, chromosome segregation defects during mitosis started only after the seventh division cycle when the residual amount of centromeric CENP-A was around 1% of the initial level (Fachinetti et al. 2013). Accordingly, successful progression through syncytial NCs after injection of transcription inhibitors does not necessarily indicate that CID loading proceeds normally, if fly centromeres harbor a comparable excess of CID beyond the level required for centromere function. Estimates of centromeric CENP-A/CID levels in fly and humans are consistent with roughly comparable amounts in the unperturbed state (Bodor et al. 2014; Bonnet et al. 2019; Lawrimore et al. 2011; Schittenhelm et al. 2010).

Here, we report the results of our characterization of the role of transcription for CID loading during early embryogenesis in *Drosophila*. We applied microscopic quantification of centromeric CID-EGFP levels after microinjection of inhibitors at concentrations that inhibit early zygotic gene transcription effectively. However, effects on CID loading were not detected.

## Results

### Centromeric CID-EGFP deposition in the presence of alpha-amanitin

**Fig. 1:**
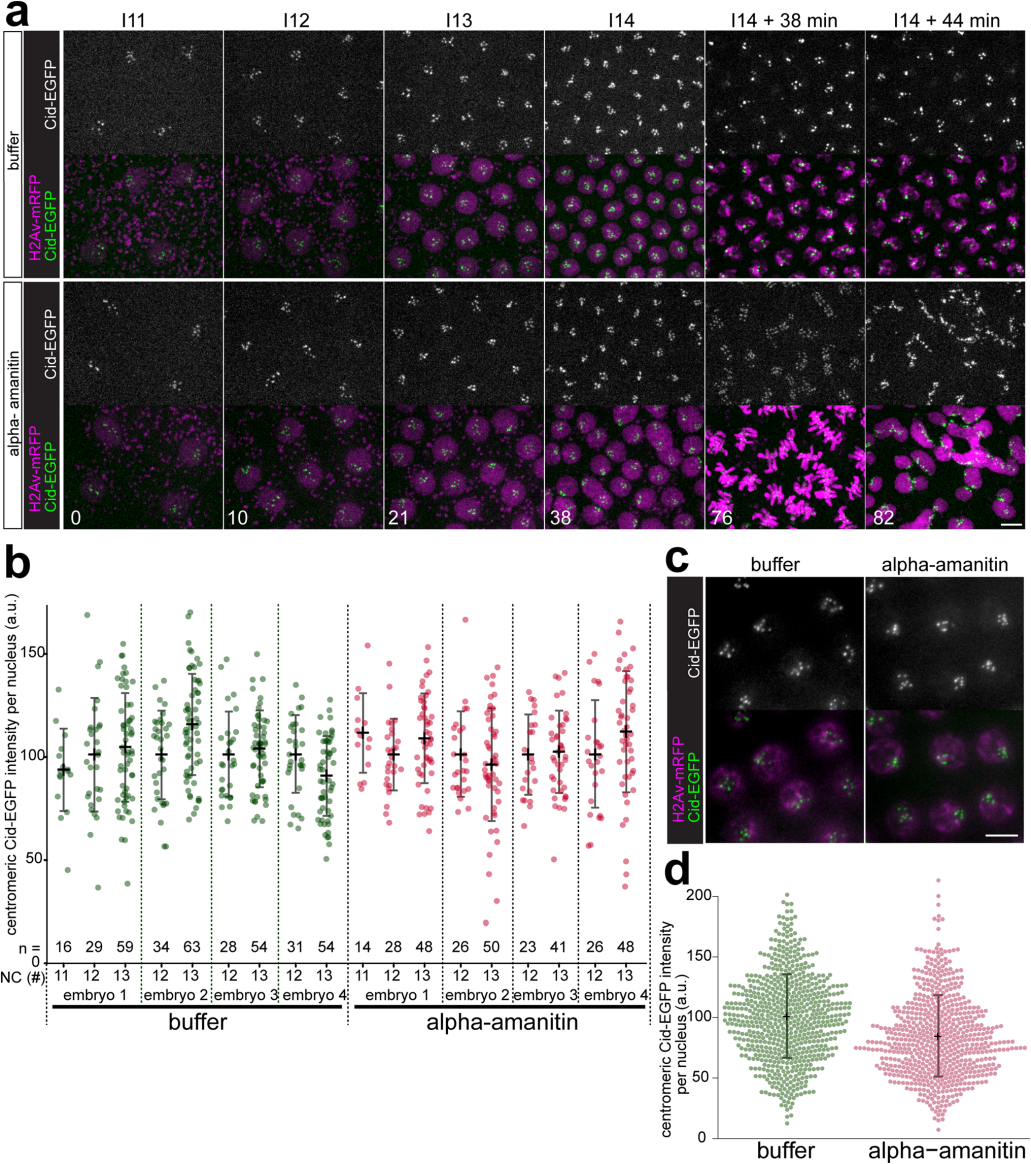
Alpha-amanitin does not preclude centromeric CID-EGFP deposition in early *Drosophila* embryos. **a–d** Embryos with CID-EGFP and His2Av-mRFP were injected before completion of NC6 with alpha-amanitin or only with buffer, as indicated. **a**, **b** Progression through NC11-14 was analyzed by time-lapse imaging. **a** Still frames illustrate CID-EGFP signals and nuclear density in early interphase (5.5 min after the metaphase to anaphase transition) during the indicated NCs. Moreover, two additional later time points from NC14 (I14 + 38 min and I14 + 44 min) reveal an abnormal synchronous and syncytial mitosis that occurs in alpha-amanitin injected embryos but not in controls. Time (min) is indicated (lower left corner). Scale bar = 5 µm. **b** Centromeric CID-EGFP signal intensities were quantified 2–3 min before anaphase onset during the indicated NCs. In addition to the values obtained for each analyzed nucleus, the number of analyzed nuclei (*n*) as well as mean and standard deviation are shown. Signal intensities observed in a given embryo were normalized by setting the mean intensity at NC12 to 100. **c**, **d** Injected embryos were aged to the stage of NC13 before fixation and imaging. **c** CID-EGFP and His2Av-mRFP signals in fixed embryos. Scale bar = 5 µm. **d** Centromeric CID-EGFP signal intensities were quantified. The values obtained for each analyzed nucleus, as well as mean and standard deviation are shown. Number of nuclei analyzed were 661 (buffer) and 633 (alpha-amanitin) from 15 embryos (buffer) and 18 embryos (alpha-amanitin), respectively

Previous analyses have clearly established that deposition of CID-EGFP at centromeres occurs during the syncytial stages of *Drosophila* embryogenesis (Schuh et al. 2007). Centromeric CID-EGFP signals increase rapidly about twofold during exit from mitosis during each nuclear blastoderm cycle. To evaluate whether transcription is required for this centromeric CID-EGFP deposition in early embryos, we performed time-lapse imaging after injection of inhibitors of transcription. In a first set of experiments, we injected alpha-amanitin, which binds and inhibits RNA polymerase (Pol) II (Wieland and Faulstich 1991). Binding of alpha-amanitin to Pol II interferes with transcript elongation; both nucleotide incorporation into nascent RNA and translocation along the template are strongly inhibited (Brueckner and Cramer 2008). Alpha-amanitin does not bind to Pol I and very high concentrations are required in the case of Pol III. We injected the inhibitor at a concentration above that was previously shown to prevent Pol II–mediated transcription in syncytial *Drosophila* embryos (Edgar and Schubiger 1986). Moreover, at this concentration, Pol I–mediated transcription is inhibited as well, indirectly via Pol II inhibition (Edgar and Schubiger 1986). The injected embryos were endowed with functional CID-EGFP derived from the maternal contribution of a transgene under control of the *cid cis*-regulatory region (Schuh et al. 2007). In addition, maternally provided histone H2Av-mRFP (His2Av-mRFP) was also present in these embryos. For control, we injected injection buffer without inhibitor. Injections were done before completion of NC6, followed by time-lapse imaging during the syncytial blastoderm stage (NC11-13). As expected (Edgar and Datar 1996; Edgar and Schubiger 1986; Gutzeit 1980), alpha-amanitin-injection did not preclude progression through NC11-NC13 (Fig. 1a, compare S1 Movie and S2 Movie). However, it prevented cellularization and the dramatic slowdown of the cell cycle, which normally occur during interphase (I) 14. As reported previously (Edgar and Datar 1996), the alpha-amanitin-injected embryos were observed to proceed after an abbreviated I14 without cellularization prematurely through mitosis (M) 14. While severely defective, this M14 was highly synchronous (Fig. 1a; S2 Movie). In contrast, cells in control embryos (Fig. 1a, S1 Movie) progressed through M14 later and asynchronously, in the normal well-known, intricate, and reproducible spatial and temporal pattern (Foe 1989). Importantly, the intensity of the centromeric CID-EGFP signals in alpha-amanitin-injected embryos during the blastoderm cycles that preceded the abnormal M14 appeared to be comparable to those displayed in buffer-injected control embryos (Fig. 1a).

For a quantitative estimation of the effect of alpha-amanitin on centromeric CID deposition during progression through the syncytial blastoderm cycles, we analyzed the intensity of centromeric CID-EGFP signals during prometaphase, a defined stage free of CID loading (Schuh et al. 2007). Based on previous analysis of CID loading in normal (non-injected) embryos with CID-EGFP and His2Av-mRFP at the syncytial blastoderm stage (Schuh et al. 2007), centromeric CID-EGFP signals per nucleus are expected to be of equal intensity in NC12 and NC13 in a given embryo, if CID loading is not affected. Normally, CID loading completely compensates the 50% reduction resulting from the partitioning of pre-existing CID onto newly replicated sister centromeres during S phase (Schuh et al. 2007). However, without any CID loading during cell cycle progression, the intensity of centromeric CID-EGFP per nucleus is predicted to be only 50% in NC13 compared to NC12. Our quantification indicated that centromeric CID-EGFP signals in NC12 and NC13 were comparable in a given embryo, not only in buffer-injected control embryos, but also after alpha-amanitin injection (Fig. 1b). On average (*n* = 4 embryos), the NC12/NC13 ratio of centromeric CID-EGFP intensity per nucleus was 1.04 (± 0.07, s.d.) after alpha-amanitin injection and 1.03 (± 0.10, s.d.) in controls, indicating that CID loading does not depend on Pol II– and Pol I–mediated transcription. Our more limited analysis of the NC11/NC12 ratio in one embryo per condition (Fig. 1b) was also in full agreement with this conclusion. Finally, monitoring centromeric CID-EGFP during progression through mitosis revealed an approximate doubling of the average signal intensities during anaphase and telophase in both alpha-amanitin-injected and control embryos (S1 Fig.), as expected based on previous analysis with uninjected control embryos (Schuh et al. 2007).

For further confirmation, we made quantitative comparisons with fixed embryos (Fig. 1c, d). Before fixation, embryos were injected either with or without alpha-amanitin. These injections were also completed before M6. After aging to I13, i.e., after progression through at least seven NCs post injection, fixation was achieved by freezing, and centromeric CID-EGFP intensities were quantified microscopically. If progression through NCs during the aging period were to occur without any deposition of new CID after alpha-amanitin injection, centromeric CID-EGFP intensities during I13 would be predicted to be only 0.8% of those in control embryos (i.e., 1/2^7^). However, intensities observed during I13 were similar in alpha-amanitin- and buffer-injected control embryos (Fig. 1c, d). Based on the apparent, limited reduction of centromeric CID-EGFP signals by 16.4%, alpha-amanitin might inhibit CID loading slightly so that 1.5% of centromeric CID is lost per NC. In conclusion, alpha-amanitin has at most a very modest inhibitory effect on CID loading during the syncytial stages.

### Centromeric CID-EGFP deposition in the presence of triptolide

For the analysis of the role of transcription for CID loading in cultured *Drosophila* cells, triptolide has been used (Bobkov et al. 2018). While alpha-amanitin interferes with elongation (Brueckner and Cramer 2008), triptolide inhibits the initiation of transcription (Bensaude 2011). Although triptolide binds to a number of cellular proteins (Tong et al. 2021), a crucial target is clearly XPB (Titov et al. 2011), a subunit of TFIIH. Triptolide binding inhibits the DNA-dependent ATPase activity of TFIIH and thereby the opening of template DNA (Bensaude 2011; Chen et al. 2015b; Henriques et al. 2013; Titov et al. 2011; Vispé et al. 2009). In analyses with *Xenopus* egg extracts, Cenp-A at kinetochores was reported to be decreased after addition of triptolide, while alpha-amanitin did not have an effect (Grenfell et al. 2016). Therefore, it appeared of interest to include experiments with triptolide into our analysis of the role of transcription for CID loading during early *Drosophila* embryogenesis.

**Fig. 2:**
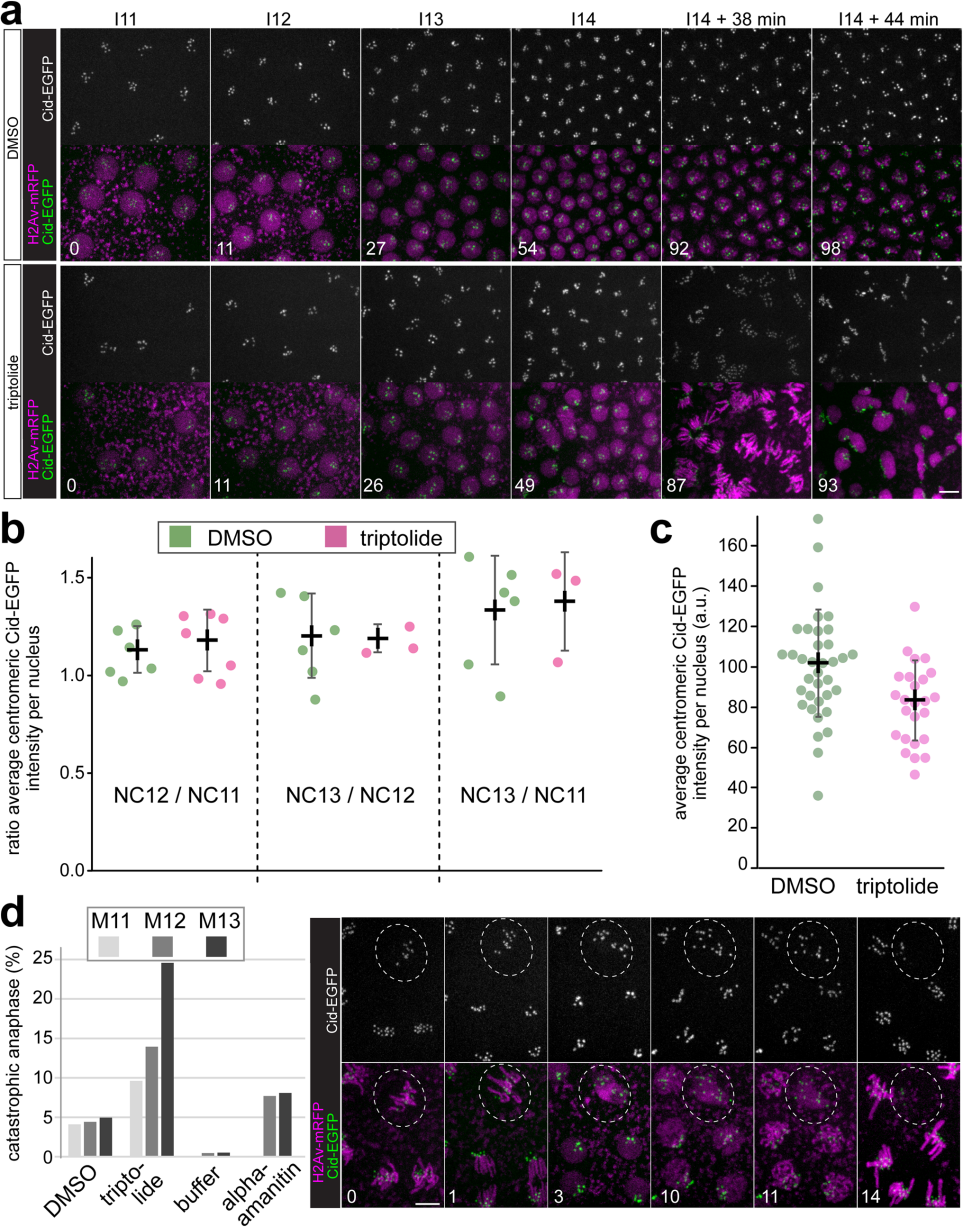
Triptolide does not preclude centromeric CID-EGFP deposition in early *Drosophila* embryos. **a–d** Embryos with CID-EGFP and His2Av-mRFP were injected before completion of NC6 with triptolide or only with DMSO, as indicated. **a**, **b** Progression through NC11-14 was analyzed by time-lapse imaging. **a** Still frames illustrate CID-EGFP signals and nuclear density in early interphase (5.5 min after the metaphase to anaphase transition) during the indicated NCs. Moreover, two additional later time points from NC14 (I14 + 38 min and I14 + 44 min) reveal an abnormal synchronous and syncytial mitosis that occurs in triptolide-injected embryos but not in controls. **b** Centromeric CID-EGFP signal intensity per nucleus was quantified 2–3 min before anaphase onset during the indicated NCs. The NC12/NC11 ratio of these intensities was determined after averaging over all analyzed nuclei of a given embryo during NC11 and NC12, respectively. The NC13/NC12 and NC13/NC11 ratios were calculated analogously. Each dot represents the ratio observed in a given embryo. The mean ratio (± s.d.) for all analyzed embryos is indicated as well. **c** Injected embryos were aged to early interphase of NC14 before fixation, imaging, and quantification of centromeric CID-EGFP signal intensity per nucleus. Dots represent the mean after averaging over all the analyzed nuclei of a given embryo (on average 117 nuclei). The mean (± s.d.) after averaging over all embryos is indicated as well. Number of embryos *n* = 36 (DMSO) and 25 (triptolide). **d** The fraction of catastrophic anaphases observed after injection of the indicated inhibitors and solvents, respectively. Injections were done before completion of NC6. Subsequent time-lapse imaging during the syncytial blastoderm stages allowed scoring during mitosis 11 (M11), 12 (M12), and 13 (M13). Still frames illustrate a catastrophic anaphase (dashed circle) in a triptolide-injected embryo. The chromosome separation failure at the end of NC11 is followed by dropping in of the affected nucleus during the following mitosis. Times (min) are indicated (lower left corner). Scale bars = 5 µm

As in the case of alpha-amanitin, we injected triptolide into embryos expressing His2Av-mRFP and CID-EGFP. For control, we injected the solvent DMSO. Injections were again completed before M6, followed by time-lapse imaging during the stages of progression through the syncytial blastoderm cycles. Similar as alpha-amanitin, triptolide did not arrest progression through the syncytial cycles (Fig. 2a), as expected (Hug et al. 2017). Moreover, we also observed an absence of cellularization during I14 and a premature progression through a synchronous and highly abnormal M14 after triptolide injection (Fig. 2a). The effect of triptolide on centromeric CID-EGFP levels was quantified as in the case of alpha-amanitin. On the one hand, quantification was done after time-lapse imaging for analysis of CID-EGFP loading in a given embryo during progression from NC11 to NC14 (Fig. 2a, b, S3 Movie and S4 Movie). On the other hand, CID-EGFP intensities were compared between populations of fixed I14 embryos injected with either triptolide or DMSO (Fig. 2c, d). Because of the greater number of embryos analyzed by time-lapse imaging after triptolide injection, data is presented in a more processed form (Fig. 2b). Rather than displaying the centromeric CID-EGFP intensity per nucleus for all the analyzed nuclei, the ratio between the mean values obtained in a given embryo in NC12 and NC11, respectively, was calculated, and also averaged over all the analyzed embryos for a given treatment. Beyond the average NC12/NC11 ratio, the average ratios NC13/NC12 and NC13/NC11 were determined for both control embryos (DMSO-injected) and triptolide-injected embryos, respectively. In control embryos, all these ratios are expected to be one, if progression through an NC is accompanied by CID-EGFP deposition to an extent that achieves constancy of the total centromeric CID level per nucleus. In contrast, if CID-EGFP deposition is completely inhibited by triptolide, the NC12/NC11 and NC13/NC12 ratios are expected to be 0.5 and 0.25 in the case of the NC13/NC11 ratio, assuming that pre-existing centromeric CID-EGFP is still distributed quantitatively onto sister centromeres without any loss. The ratios observed in triptolide-injected embryos were very similar to those obtained from control embryos (Fig. 2b), indicating that triptolide does not inhibit CID-EGFP deposition during progression through the syncytial blastoderm NCs. We note that in both control and triptolide-injected embryos, the ratios were slightly higher than one (Fig. 2b), suggesting that progression through the syncytial blastoderm NCs might be accompanied by a modest increase in centromeric CID levels. The comparison of the average centromeric CID-EGFP intensities in populations of fixed early NC14 embryos after early injection with either DMSO or triptolide did also not reveal a substantial difference (Fig. 2d). Based on the apparent, limited reduction of centromeric CID-EGFP signals by 18% after progression through at least eight NCs after injection, triptolide might inhibit CID loading slightly so that 1.5% of centromeric CID is lost per NC compared to controls.

In conclusion, the results obtained after triptolide injection were essentially identical to those after alpha-amanitin injection. Triptolide did not inhibit centromeric deposition of CID-EGFP during progression through the NCs of the syncytial stages, or at most to a very minor extent.

As we are not aware of previous time-lapse imaging of triptolide effects on early embryonic development, we point out that progression through the syncytial blastoderm NCs was not entirely normal after injection of this inhibitor. A noticeable number of nuclei failed to separate chromosomes successfully during anaphase. In the following NC, the affected nuclei dropped into the interior of the embryo. Quantification of the nuclei undergoing such a catastrophic anaphase clearly confirmed that their frequency was highest after triptolide injection, increasing from M11 to M13 (Fig. 2d). Alpha-amanitin injection resulted also in some catastrophic anaphases, but at a lower frequency (Fig. 2d). In control embryos injected with either DMSO or injection buffer, anaphase defects were even less frequent (Fig. 2d). Importantly, centromeric CID-EGFP signals before catastrophic anaphases were not reduced in the affected nuclei.

**Fig. 3:**
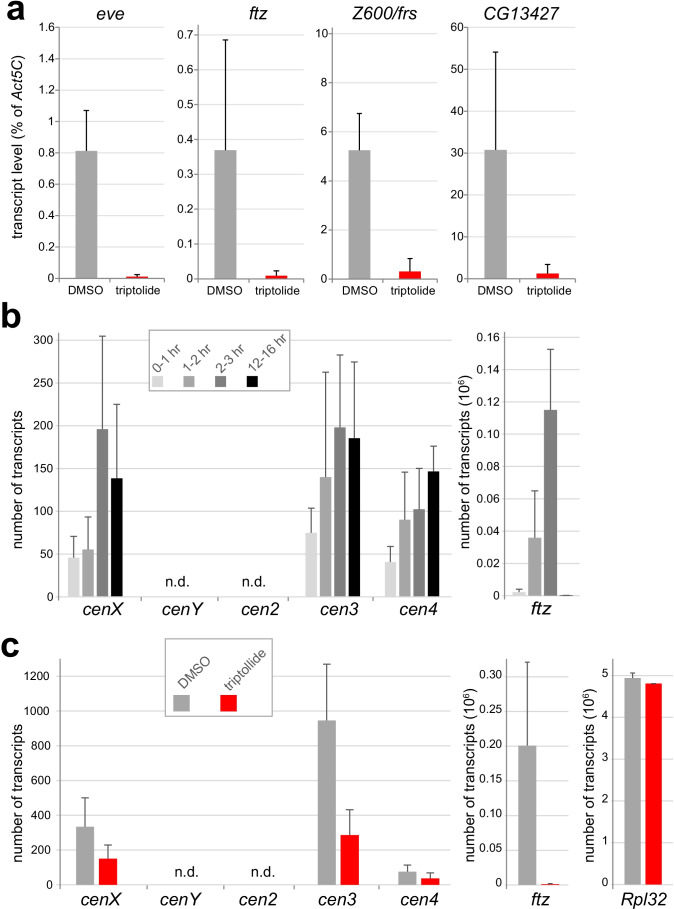
Effects of triptolide on early zygotic genes and potential centromere transcript levels. **a** Triptolide inhibits the transcription of the analyzed early zygotic genes (*eve*, *ftz*, *Z600*/*frs*, and *CG13427*). Triptolide or only the solvent DMSO were injected into embryos before NC6 completion, followed by aging to NC14, RNA isolation, and analysis by RT-qPCR. *Act5c* transcripts were analyzed as well and used as a reference for quantification of transcript levels. Mean ± s.d. (*n* = 3). **b** Level of putative centromere transcripts during embryogenesis. Embryos were collected and aged to the indicated stages before RNA isolation and analysis by RT-qPCR with primer pairs for detection of transcripts derived from G2/Jockey-3 variants reported to be uniquely present at the centromere of either chrX (*cenX*), chrY (*cenY*), chr2 (*cen2*), chr3 (*cen3*), or chr4 (*cen4*). For control, *ftz* transcripts were analyzed in parallel. *Act5c* transcripts, assumed to be present in 15 million copies per embryo, were used as a reference for an estimation of the number of transcripts present in an embryo. Mean ± s.d. (*n* = 3 in the case of 0–1 and 14–16-h AED embryos, and 4 in the case of 1–2 and 2–3-h AED embryos). n.d. = not detected. **c** Effects of triptolide on the level of potential centromere transcripts in early embryos. Triptolide or only the solvent DMSO were injected into embryos before NC6 completion, followed aging to NC14, RNA isolation, and analysis by RT-qPCR. Beyond the primer pairs for the indicated putative centromere transcripts, *ftz* and *Rpl32* transcripts were analyzed in parallel for control. *Act5c* transcripts, assumed to be present in 15 million copies per embryo, were used as a reference for an estimation of the number of transcripts present in an embryo. Mean ± s.d. (*n* = 4). n.d. = not detected

To confirm that injection of triptolide into syncytial embryos inhibits transcription as expected, we analyzed transcript levels of selected genes using reverse transcription quantitative real-time polymerase chain reaction (RT-qPCR). The genes selected for analysis (*eve*, *ftz*, *Z600*/*frs*, and *CG13427*) are known to be transcriptionally activated during the syncytial blastoderm stages with peak activity at the cellular blastoderm stage. Moreover, maternally derived transcripts of these genes are absent in embryos (Pilot et al. 2006). In contrast, maternal transcripts are highly abundant in embryos in the case of *Act5C*, a gene characterized by delayed activation of zygotic transcription during late cellular blastoderm. As a result, at most, 5% of the total *Act5C* transcript level present during NC14 are contributed by zygotic transcription (Lott et al. 2011; Pilot et al. 2006). Accordingly, if transcription is indeed inhibited by triptolide, its injection into early embryos precludes subsequent accumulation of transcripts from zygotic genes, with negligible effects on *Act5c* mRNA levels. Thus, *Act5c* transcripts were used as a reference for quantification of transcripts from the selected zygotic genes at the time of NC14 after injection of either triptolide or DMSO prior to completion of NC6. Triptolide reduced the transcripts of *eve*, *ftz*, *Z600*/*frs*, and *CG13427* to 1.4, 2.9, 6.8, and 2.5% of the levels observed in the DMSO controls (Fig. 3a). We conclude that injection of triptolide into early embryos inhibits zygotic transcription very effectively.

The recent molecular characterization of *Drosophila* centromeres (Chang et al. 2019) has revealed an enrichment of G2/Jockey-3, a non-LTR retroelement, in centromeric DNA sequences that are CID-associated according to chromatin immunoprecipitation and sequencing (ChIP-Seq). Moreover, distinct sequence variants of G2/Jockey-3 uniquely present in the centromere of only one of the five chromosomes (chr) were reported to be detectable by specific primer pairs. With such primer pairs targeting G2/Jockey-3 variants reported to be uniquely present at the centromere of chrX, chr3, or chr4, evidence for the presence of centromeric transcripts in embryos was obtained by RT-qPCR (Chang et al. 2019). In the case of chrX and chr2, the primer pairs targeting such unique centromere-specific variants did not detect transcripts. The RNA samples analyzed in these experiments were isolated from embryos collected overnight (Chang et al. 2019). We repeated the RT-qPCR analyses with shorter embryo collections that were aged to different developmental stages in order to evaluate developmental regulation of the reported putative centromeric transcripts. In particular, we analyzed whether these transcripts are also present during the early embryonic, syncytial NCs. Thus, we analyzed samples from embryos between (a) 0–1, (b) 1–2, (c) 2–3, and (d) 12–16 h AED. Sample (a) contained embryos before zygotic genome activation (ZGA) (Kwasnieski et al. 2019; Vastenhouw et al. 2019). Sample (b) covered the syncytial blastoderm stages, in which a limited number of genes is transcriptionally activated during the initial minor wave of ZGA. Embryos of sample (c) were at the cellular blastoderm stage during the major wave of ZGA, which affects thousands of genes. Finally, sample (d) was from embryos, in which almost all cells were post-mitotic and during terminal differentiation. For these RT-qPCR experiments, *Act5C* transcripts were again used as a reference, and the obtained cycle threshold (Ct) values were used to estimate the number of transcripts per embryo, assuming that the number of mRNA copies in case of *Act5C* is 15 million (see discussion). Analysis of the samples (a–d) with *ftz*-specific primers revealed the expected transcriptional activation for the strong transient peak of maximal *ftz* expression in early embryos, confirming the quality of our timed embryo collections (Fig. 3b).

With 12–16-h embryos, we obtained RT-qPCR results for the putative centromere transcripts (Fig. 3b) very similar to those reported previously for embryos collected overnight (Chang et al. 2019). While the primer pairs for the centromeric target sequences on chrY (*cenY*) and chr2 (*cen2*) failed to yield products, those targeting chrX (*cenX*), chr3 (*cen3*), and chr4 (*cen4*) clearly detected transcripts. The level of these transcripts was very low. However, control experiments, in which reverse transcriptase was omitted, indicated that the corresponding RT-qPCR products were not derived from genomic DNA contamination. Moreover, a primer pair previously used for control (Chang et al. 2019), as it targets *Mst84Da*, which is not expressed in early embryos, did also not amplify any products from our cDNA preparations, further arguing against genomic DNA contamination. We emphasize that the apparent, very low levels of the putative centromere transcripts are at the limit of reliable detection by standard RT-qPCR assays (Taylor et al. 2019), calling for cautious interpretation (see discussion).

In 0–1-h embryos, the levels of the putative centromere transcripts of *cenX*, *cen3*, and *cen4* were even lower compared to 12–16-h embryos (Fig. 3b). Interestingly, however, the level of these transcripts appeared to increase during syncytial blastoderm (1–2-h embryos) and cellular blastoderm (2–3-h embryos) (Fig. 3b). In the case of *cenX*, the difference between the mean DeltaCt value of 2–3-h embryos and that of the preceding stages (0–1- and 1–2-h embryos) reached statistical significance (*p* = 0.03, *t* test).

In summary, our developmental analysis of putative centromeric transcripts during embryogenesis (Fig. 3b) argued that very low levels of these transcripts are present during the earliest embryonic stages. These transcripts might be of maternal origin. Alternatively, zygotic transcription of centromeres might start very early in embryogenesis. In any case, zygotic transcription seems to augment the amount of these transcripts during progression through the syncytial and cellular blastoderm stages.

The apparent zygotic transcription of some of the centromeric retroelements that was detectable during the syncytial and cellular blastoderm stages provided an opportunity to study whether triptolide inhibits this centromeric transcription. Therefore, we injected either triptolide or DMSO before completion of NC6 and aged the injected embryos to the stage of NC14 before RNA isolation and analysis by RT-qPCR. As a reference, *Act5C* transcripts were used again as in the preceding experiments. On average, triptolide was found to reduce the level of *ftz* mRNA, which was analyzed for positive control, to 0.5% of that observed after DMSO injection (Fig. 3c). As a negative control, we analyzed *RpL32* mRNA. Comparable to *Act5C*, very high levels of maternally derived, stable transcripts are known to be present in the case of *RpL32* and ribosomal protein genes in general (Bashirullah et al. 1999; Pilot et al. 2006). Moreover, their zygotic expression starts relatively late during embryogenesis (Lott et al. 2011). Zygotic transcripts therefore contribute marginally to the total level of *RpL32* mRNA present during NC14, and hence, triptolide injection was expected to cause at most a marginal reduction in the level of these transcripts. Indeed, the mean *RpL32* mRNA levels after triptolide injection remained high at 97% of the DMSO control value. In the case of the centromeric targets (*cenX*, *cen3*, and *cen4*), triptolide was found to decrease transcript levels to 45, 30, and 49%, respectively, compared to the DMSO controls, although statistical significance was only reached in the case of *cen3* (*p* = 0.028; *t* test). Overall, these results support the suggestion that the centromeric targets *cenX*, *cen3*, and *cen4* are transcribed during progression through the syncytial NCs and that triptolide blocks this transcription.

## Discussion

Based on analyses with cultured cells, transcription of centromeric CENP-A chromatin is thought to be required during cell proliferation for an eventual incorporation of new CENP-A nucleosomes into sister centromeres generated by DNA replication (Bobkov et al. 2018, 2020; Corless et al. 2020; Liu et al. 2021; Mellone and Fachinetti 2021). However, a dependence of centromeric CENP-A deposition on transcription is difficult to reconcile with the notion that the initial embryonic stages proceed in the absence of transcription in a wide range of animal species. Therefore, we have analyzed the role of transcription for CID loading in *Drosophila* embryos. Our results demonstrate that alpha-amanitin and triptolide, two potent inhibitors of RNA polymerase II–mediated transcription, have at most a marginal effect on centromeric CID deposition during progression through the early embryonic syncytial NCs.

While alpha-amanitin is an extensively characterized highly specific inhibitor of transcription that has also often been used for experiments with *Drosophila* embryos, triptolide is a more recently identified inhibitor. Our analysis of early zygotic genes (*eve*, *ftz*, *Z600*/*frs*, and *CG13427*) confirmed that it acts as a potent inhibitor of transcription after injection into *Drosophila* embryos. Moreover, as also observed after alpha-amanitin injection, triptolide precluded cellularization, which is known to depend on transcription of early zygotic genes and which normally occurs during interphase of NC14. Like alpha-amanitin, triptolide also resulted in a failure to slow down cell cycle progression. Instead of the normal extended interphase of NC14, a premature and synchronous entry into M14 was observed. In comparison to alpha-amanitin, however, triptolide interfered more frequently with cell cycle progression already during the syncytial blastoderm cycles NC10-13. A higher number of nuclei did not achieve normal chromosome separation during M10-13, and mitotic failure was followed by an eventual loss of the affected nuclei into the interior. These catastrophic anaphases were highly reminiscent of those described after injection of inhibitors of DNA replication or other DNA-damaging drugs (Fogarty et al. 1997; Takada et al. 2003). The stronger abnormalities during the syncytial blastoderm NCs resulting from triptolide, for which a number of target proteins have been identified (Tong et al. 2021), might reflect side effects on processes other than transcription. However, although different with regard to frequency, the characteristics of the abnormal mitoses induced by the two inhibitors were indistinguishable. Thus, it remains conceivable that triptolide simply acts as a more powerful inhibitor of transcription. Overall, the effects of alpha-amanitin and triptolide argue strongly that early zygotic transcription results in products that contribute increasingly to the robustness of cell cycle progression during the syncytial blastoderm. The massive abnormalities of the extra syncytial M14 that occurs after both alpha-amanitin and triptolide injection indicate that maternal provisions are definitely insufficient for a normal cell cycle progression by this time.

Our experiments with triptolide also provided evidence suggesting that centromeric transcription accompanies progression through the syncytial blastoderm NCs and that triptolide inhibits this centromeric transcription, although without consequences for centromeric CID deposition. We emphasize that our evidence remains preliminary. To assess centromeric transcription, we have relied on primer pairs recently suggested to target unique centromeric G2/Jockey-3 insertions within CID chromatin (Chang et al. 2019). However, as also pointed out in the original description, given that a gapless telomere to telomere genome assembly does not yet exist, it cannot be excluded that these primer pairs might detect additional copies of these G2/Jockey-3 variants present in remaining gaps that are particularly prominent in the case of DNA sequences within pericentromeric heterochromatin. In fact, in the case of the primer pairs for *cenX* and *cen3*, our BLAST searches detected two non-centromeric perfect match targets in the reference genome (r6.42) for each pair, and a target with only one base pair mismatch in the case of the *cen4* primer pair. It is therefore evident that definitive analyses of centromere transcription will require a preceding comprehensive characterization and confirmation of unique centromeric sequences in CID chromatin in the genetic background used for analysis.

An additional limitation for the quantification of the putative centromeric transcripts arose from their exceedingly low levels. The relatively high Ct values resulting in standard RT-qPCR assays are associated with increasing subsampling and other errors (Taylor et al. 2019). As a reference, we have used *Act5C* mRNA in our analyses. Our estimate of the number of *Act5C* mRNA copies per embryo is based on the absolute mRNA copy numbers in early embryos that have been reported for some genes, including *bcd*, *zld*, *hb*, and *sna* (Boettiger and Levine 2013; Little et al. 2013; Petkova et al. 2014; Sandler and Stathopoulos 2016). Using these absolute copy numbers in combination with quantitative RNA-Seq data (Graveley et al. 2011), extrapolation to Act5C indicates that an estimated 15 million mRNA copies are present in the embryo in the case of this gene, i.e., 5% of the reported number of all maternal poly(A) transcripts (Davidson 1986). Based on this admittedly approximate estimate and our data at face value, transcription during the late syncytial blastoderm cycles generates only a few hundred centromeric transcripts in the case of *cenX*, *cen3*, and *cen4*. The number of nuclei and centromeres during these stages is at least tenfold higher. However, in principle, centromeric transcripts might have a low stability and a single passage of an RNA polymerase might be sufficient for displacement of a placeholder nucleosome. Thus, future definitive analyses of centromere transcription will have to cope with potentially very low transcript levels.

While our experiments rule out a requirement of RNA polymerase II–mediated transcription for centromeric CID deposition during the syncytial NCs of early *Drosophila* embryogenesis, they have not directly addressed the possibility that centromeric CID chromatin might be transcribed by another RNA polymerase during these stages. Careful analyses after alpha-amanitin injection have indicated that this inhibitor also prevents RNA polymerase I–mediated transcription indirectly via RNA polymerase II inhibition (Edgar and Schubiger 1986). Similarly, inhibition of RNA polymerase I beyond RNA polymerase II has also been observed with triptolide in cultured mammalian cells (Vispé et al. 2009). In any case, RNA polymerase III is clearly a poor target or not affected at all by alpha-amanitin and triptolide. However, given the enormous speed of the early embryonic cell cycles with a window of only about 2 min during which centromeric CID deposition appears to be completed (Schuh et al. 2007), we consider it likely that this process does not depend on transcription at all at the start of embryogenesis.

In principle, incorporation of placeholder nucleosomes during replication of centromeric DNA might not occur during the rapid syncytial NCs. However, overall nucleosome density appears to be comparable during syncytial and later stages based on quantitative histone H3 ChIP-Seq (Li et al. 2014). The reported slightly lower nucleosome density in syncytial blastoderm embryos (Li et al. 2014) is readily explained by the absence of G phases during these early stages, resulting in a preferential analysis of S phase nuclei, in which nucleosomes are known to be displaced transiently during DNA replication fork passage. However, in contrast to nucleosome density, the overall extent of posttranslational histone modifications was found to differ dramatically in syncytial embryos at least in the case of the analyzed modifications (Li et al. 2014).

Recent analyses with cultured cells have indicated that centromeric transcription by RNA pol II in cooperation with the histone chaperone FACT does not just disassemble selectively placeholder nucleosomes but also CID/Cenp-A nucleosomes (Bobkov et al. 2020; Chen et al. 2015a; Swartz et al. 2019). While CID/Cenp-A nucleosomes appear to be re-incorporated by the transcription elongation factor and histone chaperone Spt6, placeholder nucleosomes are not. The basis for discrimination between the two types of nucleosomes by Spt6 is not understood but might involve differential posttranslational modifications. Future analyses of the roles of FACT, Spt6, or related histone chaperones, as well as of histone modifications, for centromeric CID/Cenp-A deposition during syncytial NCs should be of interest.

As progression through the syncytial cycles proceeds in the presence of alpha-amanitin and triptolide, the early *Drosophila* embryo permitted a conclusive demonstration here that centromeric CID loading can occur in the absence of RNA polymerase II activity. A definitive clarification to what extent CID loading in cultured cells and other developmental stages depends on transcription of CID chromatin by RNA polymerase II is far more difficult due to the paramount pleiotropic importance of RNA polymerase II in combination with slower cell cycles and extended periods of CID loading. However, extremely elegant experiments have provided substantial support for an involvement of RNA polymerase II in CID loading in cultured cells (Bobkov et al. 2018, 2020). Accordingly, the mechanisms of centromeric CID deposition might vary in a stage- and cell-type-specific manner. In fact, in a more general sense, this conclusion seems inescapable, as a growing number of analyses has revealed obvious cell-type-specific differences in the timing of CID deposition during the cell cycle (Carty et al. 2021; Dattoli et al. 2020; Del García et al. 2018; Dunleavy et al. 2012; Ranjan et al. 2019; Raychaudhuri et al. 2012; Schuh et al. 2007). Given the apparent mechanistic flexibility of CID loading in *Drosophila*, a wider consideration of cell types and developmental stages in other species, including mammals, may be warranted.

## Materials and methods

### Microinjection of Drosophila embryos

Egg collection and microinjection were performed following standard procedures. Briefly, eggs were collected from the line w*;* P{w*^+^*, His2Av-mRFP}* III.1, *P{w*^+^*, cid-EGFP-cid}* III.1/*TM3*, *Ser* (Schuh et al. 2007) on apple juice agar plates during about 15 min at 25 °C. For removal of the chorion, embryos were incubated for 3 min in 3% sodium hypochlorite at room temperature. After extensive rinsing with water, followed by drying for around 7 min, embryos were aligned along the edge of an agar block and transferred onto a coverslip with a strip of glue previously applied after extraction with heptane from double stick tape. Embryos were covered with halocarbon oil to prevent further drying, and microinjected thereafter. At the time of injection, embryos were between 35 and 50 min after egg deposition (AED). Thus, based on the well-known temporal program of wild-type early embryogenesis (Foe and Alberts 1983), microinjection was done before completion of NC6, except in the case of some rare embryos retained in the female and laid after having already progressed further through embryogenesis. Embryos were injected with either injection buffer (5 mM KCl, 0.1 mM sodium phosphate, pH 7.8) or with an alpha-amanitin stock solution in injection buffer (alpha-amanitin, Sigma-Aldrich A2263-1MG, 500 µg/ml). In the case of triptolide (Adipogen Life Sciences, CDX-T0237-M005), the stock solution (10 mM) was made with dimethyl sulfoxide (DMSO), and DMSO was therefore used for control injections. With an estimated dilution factor of 50 (Edgar and Schubiger 1986), the final inhibitor concentrations in the embryos were 10 µg/ml in the case of alpha-amanitin and 200 µg/ml in the case of triptolide.

### Time lapse imaging

After microinjection, embryos were aged at 25 °C for about 45 min so that imaging started 80–95 min AED. Imaging was also performed at 25 °C in a room with temperature control using a spinning disc confocal microscope (VisiScope with a Yokogawa CSU-X1 unit combined with an Olympus IX83 inverted stand and a Photometrics evolve EM 512 EMCCD camera, equipped for red/green dual channel fluorescence observation; Visitron systems, Puchheim, Germany). After injection of alpha-amanitin or injection buffer, image stacks with 19 *z*-sections spaced by 500 nm were acquired with a 100 × /1.4 oil immersion objective every 60 s using 100-ms exposure times for both EGFP and mRFP signals. By multi-position imaging, we imaged all the injected embryos on a slide that displayed a nuclear density corresponding to NC11 or NC12 at the start of imaging. Before the start of time-lapse imaging, the center of the image stack was set to the *z* plane that revealed a maximal number of CID-EGFP signals in a given embryo. Imaging was performed analogously after injection of triptolide or DMSO except that the acquired stacks comprised 30 *z*-sections.

### Imaging after fixation

Embryos were aged at 25 °C for 70 min after injection with either alpha-amanitin or injection buffer so that they were between 105 and 125 min AED at the time of fixation. In the case of injection with either triptolide or DMSO, aging before fixation was for 100 min to generate a sample with embryos between 135 and 155 min AED. For fixation, the coverslip with the injected embryos covered by halocarbon oil was transferred onto the flat surface on the back of an aluminum heat block that had been pre-cooled to − 20 °C in a freezer. The coverslip on the aluminum block was stored at − 20 °C until immediately before microscopic analysis. For microscopy, the coverslip was brought to room temperature and mounted on the microscope stage. In the case of the alpha-amanitin/buffer injection experiments, an image stack with 30 *z*-sections spaced by 250 nm and extending from the coverslip surface into the embryo interior was acquired from each embryo with a 100 × /1.4 oil immersion objective on an inverse Zeiss Cell Observer HS wide-field microscope. In the case of the triptolide/DMSO injection experiments, a 40 × /1.3 oil immersion was used for the acquisition of image stacks with 19 *z*-sections spaced by 500 nm.

### Image analysis

For analysis of time-lapse data, a constant minimal range of *z*-planes (8–12 optical sections) that comprised all of the centromeric CID-EGFP signals was selected in each imaged embryo using IMARIS software. Embryos displaying an extensive drift of centromeric CID-EGFP signals over time along the *z*-axis out of this range were discarded from analyses. Moreover, image stacks were cropped along the *x*- and *y*-axes so that only the central regions were retained, in which the embryo periphery was apposed flat against the coverslip. To select the time points for our quantification of CID-EGFP signal intensities, we first identified the first anaphase time points during each of the imaged M phases, followed by stepping back two time points. The selected time points were therefore during the prometaphase-to-metaphase transition when centromere clustering is minimal. Maximum intensity projections were generated using ImageJ. For quantification of centromeric CID-EGFP signals, an oval region of interest (ROI) including all centromeric CID-EGFP dots of a given nucleus was defined manually. Nuclei affected by catastrophic anaphase were not considered in our quantitative analyses. For background correction, the ROI size was increased by three pixels, and signals located within this larger ROI but outside of the smaller ROI were considered to reflect non-centromeric background fluorescence. The background-corrected centromeric CID-EGFP intensity per nucleus in arbitrary units (a.u.) was calculated as the difference between the integrated pixel intensity within the smaller ROI and the average pixel intensity in the background region multiplied by the pixel number of the smaller ROI area. For comparison of centromeric CID-EGFP intensity in consecutive syncytial blastoderm NCs, the values observed for all nuclei during NC12 in a given embryo were averaged. This average was set to 100 a.u. and used for normalization of all the values for background-corrected centromeric CID-EGFP intensity per nucleus that were obtained in a given embryo.

In the case of the analyses with fixed embryos, we also used IMARIS software for selection of 18 *z*-planes containing the centromeric CID-EGFP signals from the image stacks, as well as the central regions of the embryos that were apposed flat to the coverslip. Thereafter, maximum intensity projections were generated and background-corrected CID-EGFP signal intensities per nucleus were determined as described above. We considered only embryos, which displayed a number of nuclei in the imaged region that was within the range observed in non-injected control embryos at the stage of interest. After injection of either alpha-amanitin or injection buffer, we analyzed embryos with a nuclear count corresponding to that of interphase 13. After injection of either triptolide or DMSO, we analyzed those with a nuclear density corresponding to early interphase 14.

Dot plots for figures were generated online (http://shiny.chemgrid.org/boxplotr/) (Spitzer et al. 2014) and imported into Adobe Illustrator for labeling.

### RT-qPCR experiments

The embryos analyzed with RT-qPCR were collected from the line w*; *P{w*^+^*, His2Av-mRFP}* III.1, *P{w*^+^*, cid-EGFP-cid}* III.1/*TM3*, *Ser*. In the case of our analysis of triptolide effects on transcription of early zygotic genes (*eve*, *ftz*, *Z600*/*frs*, and *CG13427*), embryos were collected for 20 min before injection with either triptolide or DMSO as described above. After aging, the injected embryos (2:40–3 h AED) were gently rinsed without detaching them from the coverslip, first with heptane to remove halocarbon oil and then with isopropanol to remove the heptane. With a brush, embryos were transferred into a glass homogenizer containing 100 µl of TRIzol (Life Technologies, Ambion) and homogenized for 30 s using a motorized pestle. Thereafter, 300 µl of TRIzol and 5 µl of a glycogen stock solution (20 µg/ml) were added. After mixing, the sample was cleared by centrifugation (10 min, 12,000 × g, 4 °C). The supernatant was extracted with chloroform (80 µl). RNA was precipitated from the aqueous phase by addition of 0.2 ml of isopropanol. The RNA pellet was washed with 0.4 ml of 75% ethanol, dried and re-suspended in 30 µl diethyl pyrocarbonate (DEPC)–treated water. The Transcriptor First Strand cDNA Synthesis Kit (Roche, Cat No. 04897030001) was used for cDNA preparation with an anchored oligo (dT)18 primer and 1 μg of total RNA in a 20 μl reaction volume. To detect potential contamination of the isolated total RNA with genomic DNA, we made analogous samples except that reverse transcriptase (RT) was omitted. We used 2.5 μl of a 1:10 dilution of the cDNA + and − RT reactions and 5 μl PowerUp™ SYBR™ Green Master Mix (Applied Biosystems, Cat. No. A25742) for each RT-qPCR assay (10 μl total volume). In addition, RT-qPCR control assays without addition of template preparations were set up. The concentration of each primer was 300 nM. Primer sequences are given in S1 Table. Three technical replicates were analyzed using the QuantStudio 5 Real-Time PCR System (Applied Biosystems).

For the analysis of the amounts of putative centromeric transcripts by RT-qPCR in embryos, we used the primer pairs described by Chang et al. (2019) (S1 Table). Moreover, isolation and purification of total RNA, as well as cDNA preparation, was modified accordingly. In the case of analyses with embryos injected with either triptolide or DMSO, we proceeded up to embryo homogenization as described above. In the case of non-injected embryos, the chorion was removed after collection and aging by incubation for 3 min in 3% sodium hypochlorite at room temperature. After extensive rinsing with water, embryos were transferred into a glass homogenizer. Injected and non-injected embryos were homogenized in 300 μl of TRI reagent (Zymo Research, Cat No. R2050-1–200) during 30 s using a motorized pestle. After clearing homogenates by centrifugation (10 min, 12,000 × g, 4 °C), the Direct-Zol RNA miniprep plus kit (Zymo Research, Cat No. R2071) was used for isolation of total RNA from the supernatant. Turbo DNase treatment (Invitrogen, Cat No. AM2238) followed by RNeasy MinElute Cleanup Kit (50) (Qiagen, Cat. No. 74204) were used to remove DNA contamination and for RNA purification. DNase treatment followed by RNA cleanup was repeated three times. The iScript Select cDNA Synthesis kit (Bio-Rad, Cat. No. 1708896) was used for cDNA preparation with random primers from up to 1 μg of total RNA in a 20 μl reaction volume. For control, − RT reactions were also set up. RT-qPCR assays were set up as described above.

## Supplementary Information

Below is the link to the electronic supplementary material.

Supplementary Fig. 1: Centromeric CID-EGFP loading during exit from M12. Before time-lapse imaging, embryos expressing CID-EGFP and His2Av-mRFP were injected prior to completion of NC6 with either (a) only buffer or with (b) alpha-amanitin. (a) Still frames illustrate progression through M12. Time (min) given relative to t0 = first anaphase frame. Scale bar = 5 µM. The graph displays centromeric CID-EGFP signal intensities. Each dot represents the centromeric CID-EGFP signal intensity in a given nucleus (see dashed yellow circle for example). At the first five time points, each analyzed nucleus contains chromosomes with two sister centromeres. In contrast, at the last four time points, each analyzed nucleus contains chromosomes with only one centromere. Therefore, to arrive at an estimate for the average centromeric CID-EGFP level per centromere, values from nuclei at the first five time points were divided by two. Mean (red cross) and s.d., n = 20 nuclei for each time point. (b) Analogous graph.
Supplementary file1 (PDF 2655 KB)

Supplementary Movie 1: Progression through the syncytial blastoderm NCs was analyzed by time-lapse imaging after microinjection of injection buffer into an embryo expressing CID-EGFP (green) and His2Av-mRFP (white). Movie starts in interphase of NC11 and ends after ventral furrow formation during interphase of NC14. Time (min:sec) is indicated.
Supplementary file2 (MP4 4658 KB)

Supplementary Movie 2: Progression through the syncytial blastoderm NCs was analyzed by time-lapse imaging after microinjection of alpha-amanitin into an embryo expressing CID-EGFP (green) and His2Av-mRFP (white). Movie starts in interphase of NC11 and ends after progression through a premature, syncytial, synchronous and highly abnormal mitosis 14. Time (min:sec) is indicated.
Supplementary file3 (MP4 4613 KB)

Supplementary Movie 3: Progression through the syncytial blastoderm NCs was analyzed by time-lapse imaging after microinjection of DMSO into an embryo expressing CID-EGFP (green) and His2Av-mRFP (white). Movie starts in interphase of NC11 and ends during interphase of NC14. Time (min:sec) is indicated.
Supplementary file4 (MP4 3179 KB)

Supplementary Movie 4: Progression through the syncytial blastoderm NCs was analyzed by time-lapse imaging after microinjection of triptolide into an embryo expressing CID-EGFP (green) and His2Av-mRFP (white). Movie starts in interphase of NC11 and ends after progression through a premature, syncytial, synchronous and highly abnormal mitosis 14. Time (min:sec) is indicated.
Supplementary file5 (MP4 3782 KB)

Supplementary Table 1: Oligonucleotide sequences.
Supplementary file6 (XLSX 11 KB)

Supplementary Table 2: Source data.
Supplementary file7 (XLSX 295 KB)
