# Supplementary figures and images for: Incorporation of CENP-A/CID into centromeres during early Drosophila embryogenesis does not require RNA polymerase II–mediated transcription
Source: Chromosoma. 2022 Jan 11;131(1-2):1–17. doi: 10.1007/s00412-022-00767-2 (PMC9079035; doi:10.1007/s00412-022-00767-2)

**a** buffer

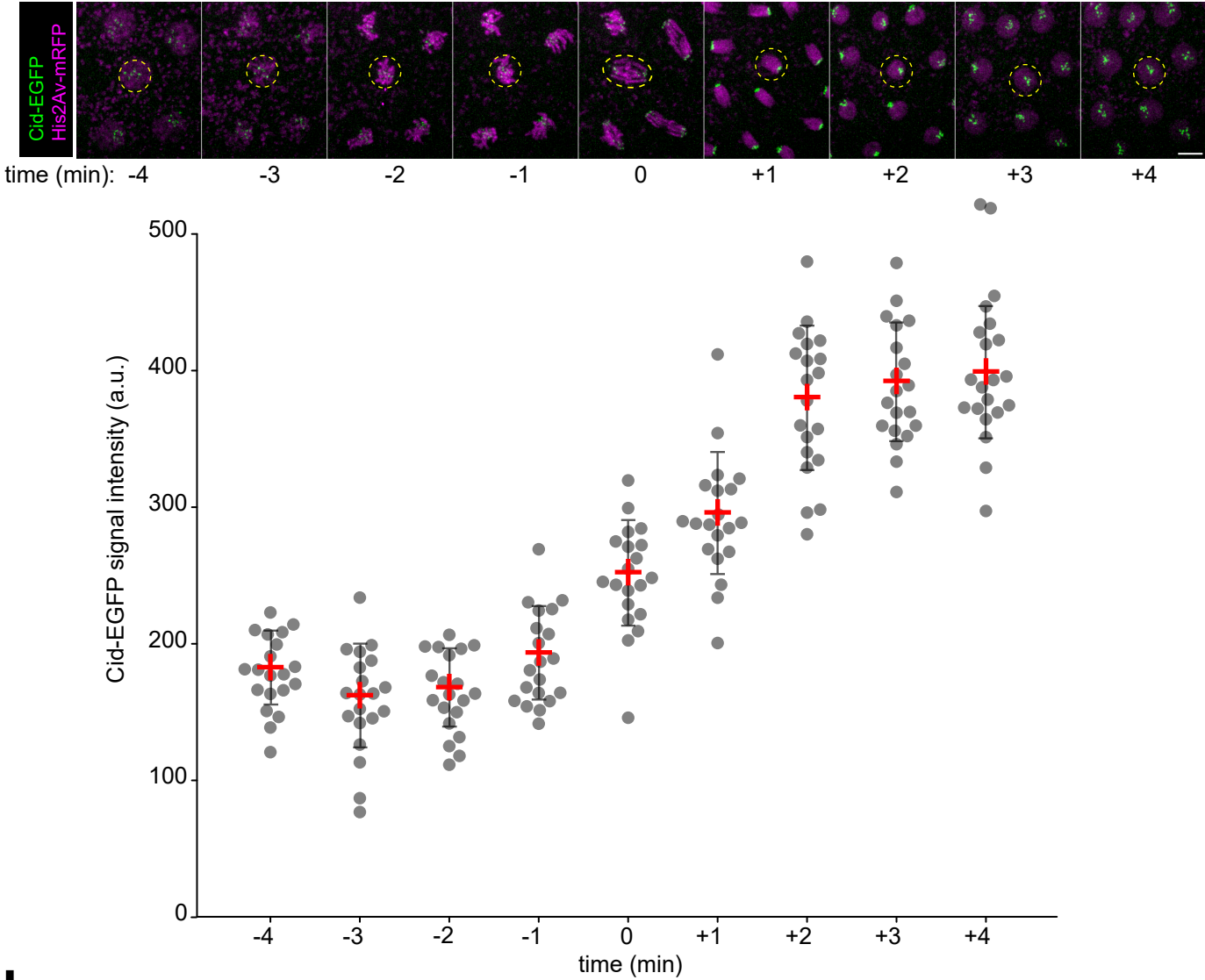

**b** alpha-amanitin

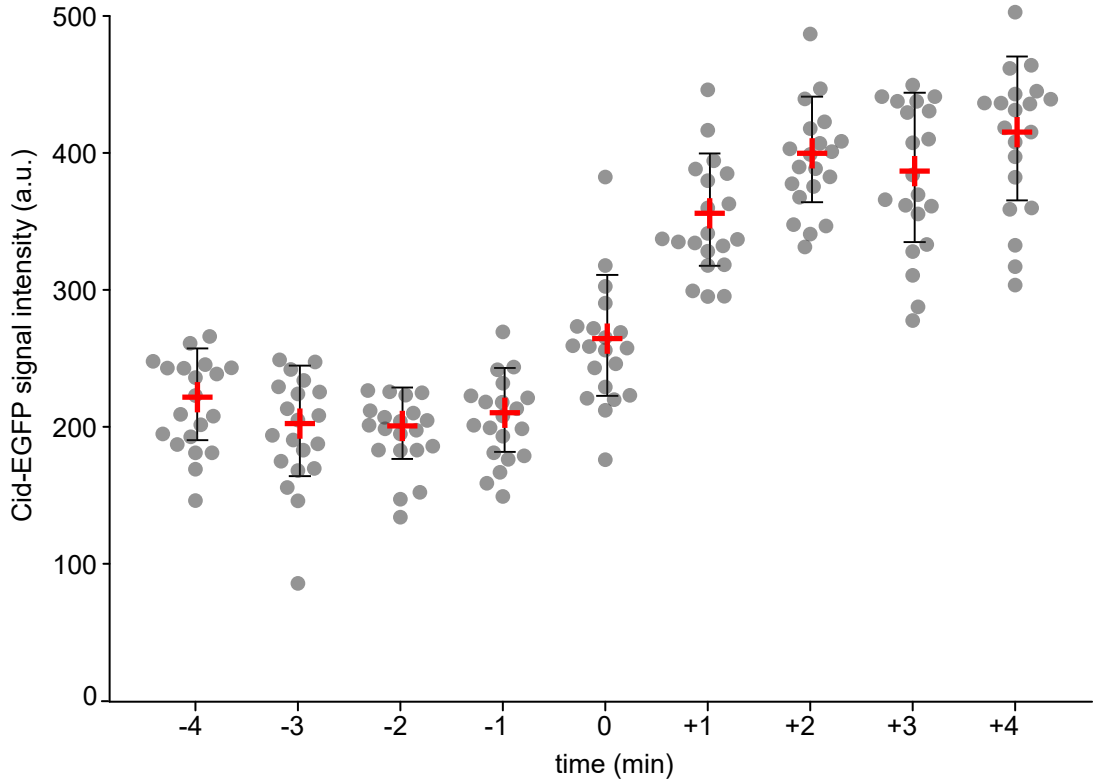

Supplement: Supplementary file 1 — Supplementary Fig. 1: Centromeric CID-EGFP loading during exit from M12. Before time-lapse imaging, embryos expressing CID-EGFP and His2Av-mRFP were injected prior to completion of NC6 with either (a) only buffer or with (b) alpha-amanitin. (a) Still frames illustrate progression through M12. Time (min) given relative to t0 = first anaphase frame. Scale bar = 5 µM. The graph displays centromeric CID-EGFP signal intensities. Each dot represents the centromeric CID-EGFP signal intensity in a given nucleus (see dashed yellow circle for example). At the first five time points, each analyzed nucleus contains chromosomes with two sister centromeres. In contrast, at the last four time points, each analyzed nucleus contains chromosomes with only one centromere. Therefore, to arrive at an estimate for the average centromeric CID-EGFP level per centromere, values from nuclei at the first five time points were divided by two. Mean (red cross) and s.d., n = 20 nuclei for each time point. (b) Analogous graph. Supplementary file1 (PDF 2655 KB) [file 412_2022_767_MOESM1_ESM.pdf]
